# Fabrication of PDMS@Fe_3_O_4_/MS Composite Materials and Its Application for Oil-Water Separation

**DOI:** 10.3390/ma15010115

**Published:** 2021-12-24

**Authors:** Jiaqi Wang, Zhenzhong Fan, Qingwang Liu, Qilei Tong, Biao Wang

**Affiliations:** School of Petroleum Engineering, Northeast Petroleum University, Daqing 163318, China; 18951355766@163.com (J.W.); neputongqilei@163.com (Q.T.)

**Keywords:** melamine sponge, superhydrophobic, magnetic, lipophilic, oil-water separation

## Abstract

The discharge of oily wastewater and oil spills at sea are the current difficulties in water pollution control. This problem often leads to terrible disasters. Therefore, the effective realization of oil-water separation is a very challenging problem. Superhydrophobic sponge is a promising oil-absorbing material. In this article, we reported a superhydrophobic sponge with nano-Fe_3_O_4_ for oil-water separation. The addition of nano-Fe_3_O_4_ allows the sponge to be recycled under the action of magnetic force. The sponge has the advantages of low cost, simple preparation and efficient oil-water separation. This kind of sponge is very worthy of promotion for the treatment of oily wastewater and marine oil spill accidents.

## 1. Introduction

Behind today’s rapid social and economic development is a large amount of industrial production and oily wastewater discharged from daily life [1,2,3]. At the same time, marine oil spills occur frequently [4,5,6]. These conditions will cause serious environmental problems during oil production [7,8]. As people’s awareness of protecting the ecological environment has gradually increased, the treatment of oily wastewater and marine oil spills has become more and more urgent. The realization of oil-water separation is the most fundamental measure to solve oil pollution to protect the environment and reduce economic waste [9,10,11]. Traditional oil-water separation methods mainly include flocculation [12], chemical degradation [13], biological filtration [14,15], etc. However, these methods have exposed the shortcomings of low separation efficiency, poor recyclability, and insufficient environmental protection in the face of increasingly severe oil pollution problems. Therefore, it is necessary to develop a new type of superhydrophobic material to achieve efficient and environmentally friendly oil-water separation.

The dip coating method has the characteristics of economy, environmental protection, high efficiency and simplicity. It is a hotspot method used in the preparation of hydrophobic and oil-absorbing materials in recent years [16,17]. The characteristics of low production cost and high porosity of modified oil-absorbing sponge also make people pay more attention to its oil-water separation ability [18]. Therefore, the preparation of superhydrophobic sponges by dipping method has attracted great interest. Modified polyurethane sponge has been widely used for oil-water separation. Lu [19] prepared superhydrophobic PDMS/ polyurethane sponge based on polyurethane sponge, but the preparation process requires the use of chromic acid solution which is harmful to the human body and the environment. Yang [20] prepared superhydrophobic/nanosilver polyurethane sponge, but the preparation process is cumbersome and costly. This shortcoming limits its application potential. In addition, polyurethane sponge has the disadvantages of flammability and low open cell rate. Last but not least, the polyurethane sponge will produce toxic smoke when it burns. These two shortcomings determine that the polyurethane sponge cannot achieve the purpose of environmental protection and high efficiency in the application of oil-water separation [21]. Melamine sponge(MS)is a low-density material formed by foaming after the reaction of formaldehyde-melamine copolymer. It has a three-dimensional porous structure with a porosity of more than 99% and does not produce toxic substances when burned. It exhibits excellent chemical stability and environmental protection features. The excellent chemical stability and environmental protection characteristics of melamine sponge make it an excellent adsorption carrier [22,23]. It must be noted that melamine sponge is naturally amphiphilic. There is nothing doubt that the melamine sponge can be used for oil-water separation only after being hydrophobically modified.

Considering the above advantages, this article chooses to use MS to prepare superhydrophobic sponge. The polydopamine coating [24,25] is formed by the oxidative polymerization of dopamine hydrochloride. Polydopamine has excellent adhesion [26] and can act as a “glue” for adhering nano-Fe_3_O_4_ and PDMS during the preparation process. The reason for choosing to add nano-Fe_3_O_4_ is to allow the sponge to recycle with the help of magnetic force after the oil-water separation work is finished. Recycling can reduce costs. PDMS can give MS excellent hydrophobic properties. With the method of dipping method, we finally obtain superhydrophobic melamine sponge (PDMS@Fe_3_O_4_/MS). The preparation process has low cost and simple operation which is more suitable for popularization and application.

## 2. Experimental Materials and Methods

### 2.1. Experimental Materials

Melamine sponge (Suzhou Huanxi Electronic Materials Co., Ltd., Suzhou, China), nano-Fe_3_O_4_ (20nm 99.0% metals basis, Aladdin Reagent Co., Ltd., Shanghai, China), absolute ethanol (Tianjin Kaitong Chemical Reagent Co., Ltd., Tianjin, China), dopamine hydrochloride (Shanghai Maclean Biochemical Technology Co., Ltd., Shanghai, China), cyclohexane (AR, Tianjin Komeo Chemical Reagent Co., Ltd., Tianjin, China), PDMS (Polydimethylsiloxane, Dow Corning, Midland, MI, USA), curing agent (Dow Corning).

### 2.2. Instrumentation and Characterization

ES type precision electronic balance (Xiamen Lai De Scientific Instrument Co., Ltd., Xiamen, China); SDC-200S type contact angle measuring instrument (Kunshan Shengding Industrial Intelligent Technology Co., Ltd., Suzhou, China); BILON3-120A Ultrasonic Cleaner (Shanghai Birang Instrument Manufacturing Co., Ltd., Shanghai, China); HJ-1 type magnetic heating and stirring (Shanghai Yuezhong Instrument Equipment Co., Ltd., Shanghai, China); DZF-6030 vacuum drying oven (Suzhou Namirui Electronic Technology Co., Ltd., Suzhou, China); Apreo-2 Field Emission Scanning Electron Microscope (Beijing Oubotong Optical Technology Co., Ltd., Beijing, China); Ultima IV X-ray diffractometer (Rigaku, Takatsuki, Osaka Prefecture, Japan); INVENIO R Fourier transform infrared spectrometer (Bruker Optics, Karlsruhe, Germany).

### 2.3. Preparation of PDMS@Fe_3_O_4_/MS

(1) Pretreatment of MS surface.

**Figure 1:**
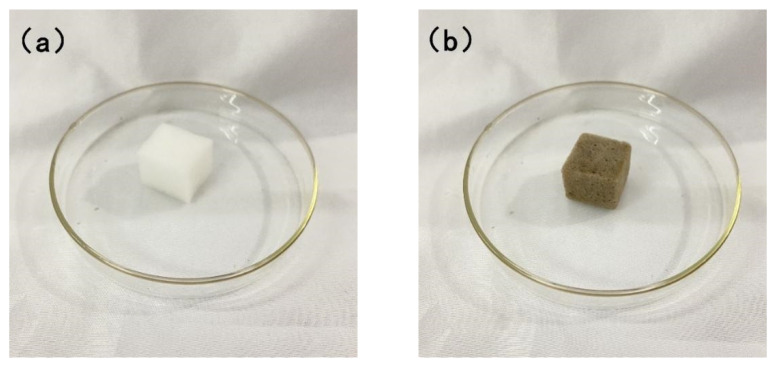
(**a**) MS and (**b**) PDMS@Fe_3_O_4_/MS.

MS (20 mm × 20 mm × 20 mm) was cleaned twice with absolute ethanol and deionized water. Then put it in an air-drying oven at 45 °C for 6 h. Take it out after drying. The washed MS is shown in Figure 1a.

(2) Polydopamine coating covers the MS skeleton.

**Figure 2:**
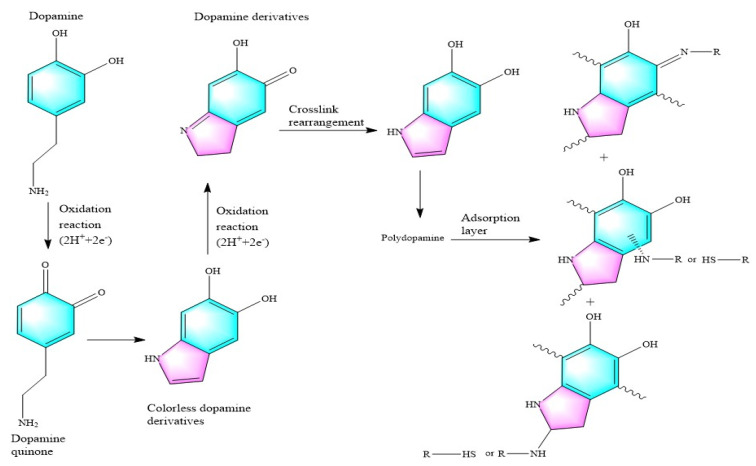
Schematic diagram of the oxidative polymerization mechanism of dopamine hydrochloride.

Weigh 240 mg of dopamine hydrochloride powder and dissolve it into 60 mL of water to prepare a dopamine hydrochloride solution with a concentration of 4 mg/mL. Put the sponge into the dopamine hydrochloride solution, so that the inside of the MS is completely infiltrated by the dopamine hydrochloride solution. Then use a magnetic stirrer to stir for 1 h. Take out the MS and let it stand in the air for 24 h to allow the dopamine hydrochloride to fully oxidize and polymerize. The polymerization mechanism of dopamine hydrochloride is shown in Figure 2.

(3) Sponge adhesion nano-Fe_3_O_4_.

Weigh 0.1 g of nano-Fe_3_O_4_ and pour it into a certain amount of ethanol solution to prepare a suspension. Then put the sponge into the solution and disperse ultrasonically for 1 h. After the sonication is completed, the sponge is placed in a constant temperature drying oven at 85 °C. Wait for the sponge to dry and take it out for later use.

(4) PDMS treats sponge superhydrophobicity.

Manually stir a mixed solution of 1 g PDMS, 20 mL cyclohexane solution, and 0.1 g hardener for 1 min. Then the sponge is completely immersed in the configured solution, placed in a constant temperature drying box, and dried at 85 °C for 12 h. After PDMS@Fe_3_O_4_/MS is completely dried, take it out and cool it to room temperature, and then soak and clean it with absolute ethanol 3 times. After cleaning, let it dry and acquire PDMS@Fe_3_O_4_/MS, as shown in Figure 1b.

### 2.4. Calculation of Adsorption Capacity and Oil-Water Separation Efficiency

#### 2.4.1. PDMS@Fe_3_O_4_/MS Adsorption Capacity Calculation

The absorption capacity of PDMS@Fe_3_O_4_/MS to oil is an important factor affecting its oil-water separation efficiency. In order to test the maximum oil absorption capacity of PDMS@Fe_3_O_4_/MS, we decided to use kerosene, 0#diesel oil and corn germ oil to test its adsorption capacity. Weigh 10 g of each oil product, and put PDMS@Fe_3_O_4_/MS into it. Take out the sponge after adsorption for 1 min. The calculation formula of adsorption capacity is as formula (1):

(1) $$Q_{f}=\frac{m_{0}-m}{m}$$

In formula (1): Q*_f_* is the adsorption capacity, g/g; m is the mass of the PDMS@Fe_3_O_4_/MS, g; m_0_ is the mass of the PDMS@Fe_3_O_4_/MS after oil absorption, g.

#### 2.4.2. PDMS@Fe_3_O_4_/MS Oil-Water Separation Efficiency Calculation

The oil-water separation efficiency is a pivotal indicator to determine whether the modified sponge has pretty performance in treating oily wastewater. Weigh 5 g of edible oil, 0#diesel oil and kerosene and mix with 50 mL of water to prepare an oil-water mixture. Put PDMS@Fe_3_O_4_/MS into the three oil-water mixtures for oil-water separation test. Finally, the method of weighing is used to calculate the oil-water separation efficiency. The oil-water separation efficiency calculation formula is as formula (2):

(2) $$R=\frac{C_{1}-C_{2}}{C}\times 100\%$$

In formula (2): R is the oil-water separation efficiency, %; *C* is the initial mass of the oil phase, g; *C*_1_ is the mass of the oil-water mixture before separation, g; *C*_2_ is the mass of the oil-water mixture after separation, g.

## 3. Results and Discussion

### 3.1. Characterization and Analysis of Original Sponge and Modified Sponge

**Figure 3:**
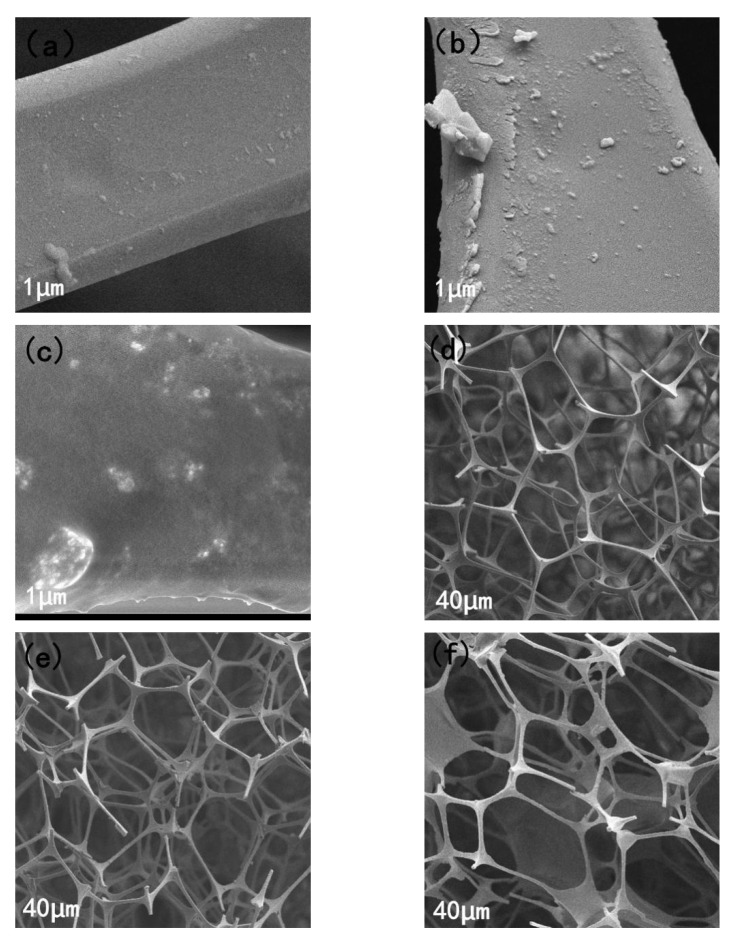
(**a**–**c**) are the scanning electron micrographs of MS, Fe_3_O_4_/MS and PDMS@Fe_3_O_4_/MS 1 µm and (**d**–**f**) are MS, Fe_3_O_4_ /MS and PDMS@Fe_3_O_4_/MS 40 µm scanning electron micrograph.

Figure 3a–c are scanning electron micrographs of MS, Fe_3_O_4_/MS and PDMS@Fe_3_O_4_/MS 1 µm. Observing the picture (a), it can be found that the surface of the MS skeleton is smooth and flat. On the other hand, MS itself contains a large amount of hydrophilic amino groups, so MS cannot be used for oil-water separation. In Figure 3b, we can find that some substances are clearly attached to the Fe_3_O_4_/MS framework. The attachment is nano-Fe_3_O_4_, which is the most important thing to realize sponge recovery. The surface of PDMS@Fe_3_O_4_/MS in Figure 3c becomes rough, and the PDMS coverage makes the sponge have an excellent hydrophobic layer. This change greatly improves the hydrophobicity of the sponge. Figure 3d–f are the scanning electron micrographs of MS, Fe_3_O_4_/MS and PDMS@Fe_3_O_4_/MS 40 µm, respectively. The three pictures all reflect the inherent three-dimensional interconnection network, providing a large number of open pores. These abundant natural pores in the MS can provide more active sites for the adsorption of the target oil, which provides a great structural basis for oil-water separation.

**Figure 4:**
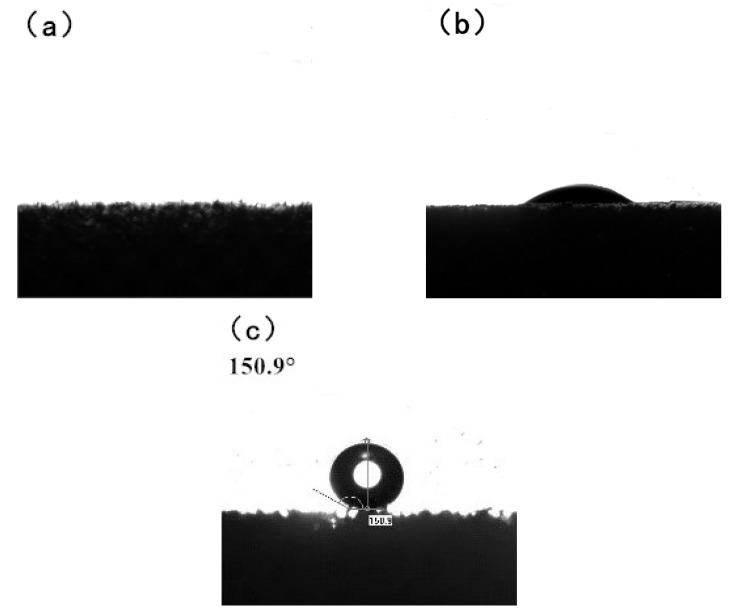
(**a**) MS water contact angle image, (**b**) Fe_3_O_4_/MS water contact angle image and (**c**) PDMS@Fe_3_O_4_/MS water contact angle image.

Figure 4 shows the water contact angle test of the melamine sponge before and after treatment. Figure 4a is an image of MS water contact angle. The water droplet is sucked in immediately after contact with the sponge. The contact angle is 0°, showing a superhydrophilic state. Figure 4b is the water contact angle image of Fe_3_O_4_/MS. From the image, we can find that the water droplets are not completely absorbed by the sponge. This phenomenon indicates that Fe_3_O_4_/MS has improved hydrophobicity compared with MS. However, this hydrophobic ability is far from reaching the standard of superhydrophobicity. Figure 4c is an image of the water contact angle on the surface of PDMS@Fe_3_O_4_/MS prepared by the modification treatment. The water droplets are attached to the surface of the sponge in a spherical form, the water contact angle is 150.9°. A water contact angle of more than 150° indicates that the material has reached a superhydrophobic state. The superhydrophobic state ensures the feasibility of PDMS@Fe_3_O_4_/MS in the fields of oil absorption and oil-water separation.

**Figure 5:**
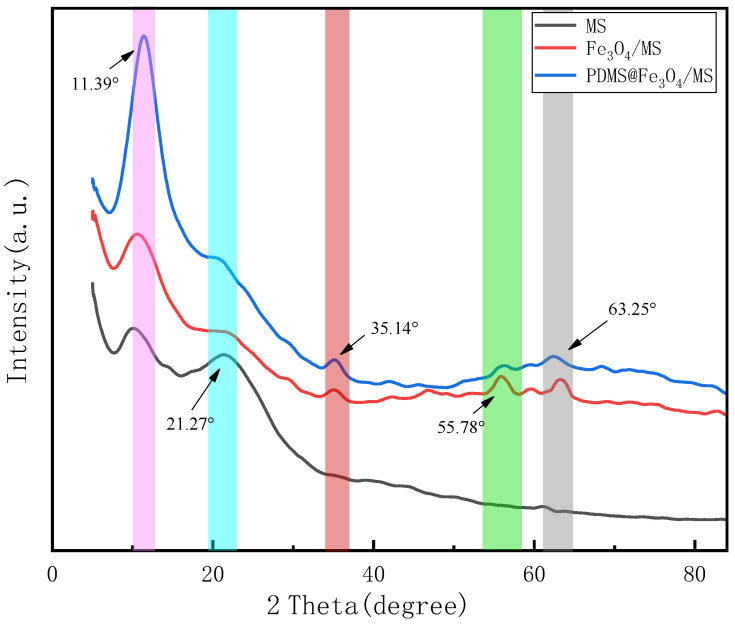
XRD patterns of MS, Fe_3_O_4_/MS and PDMS@Fe_3_O_4_/MS.

In order to prove the existence of nano-Fe_3_O_4_ and PDMS crystal structure on the surface of the sponge, X-ray diffraction (XRD) test was performed on it. The XRD test result is shown in Figure 5. In the XRD pattern of PDMS@Fe_3_O_4_/MS, the diffraction peak with a 2θ of 11.39° is attributed to the characteristic diffraction peak of the PDMS crystal plane [27], indicating that the PDMS coating is well covered on the sponge skeleton. In the MS XRD diagram, the 2θ of 21.27° is the characteristic peak of MS, which is consistent with the standard card JCPDS#24-1923. However, the XRD patterns of Fe_3_O_4_/MS and PDMS@Fe_3_O_4_/MS at 2θ of 21.27° are smooth. This phenomenon indicates that the characteristic diffraction peaks of MS are covered by nano-Fe_3_O_4_ and PDMS. The characteristic diffraction peaks of nano-Fe_3_O_4_ appear near 35.14°, 55.78° and 63.25°. This is strong evidence that the nano-Fe_3_O_4_ successfully adhered to the sponge skeleton besides the characteristic diffraction peaks of MS were masked. The characteristic diffraction peak of nano-Fe_3_O_4_ with a 2θ of 35.14° is consistent with the peak of the standard card JCPDF#19-0629. MS has no characteristic diffraction peaks near 35.14°, 55.78°, and 63.25°, which also verifies this point.

**Figure 6:**
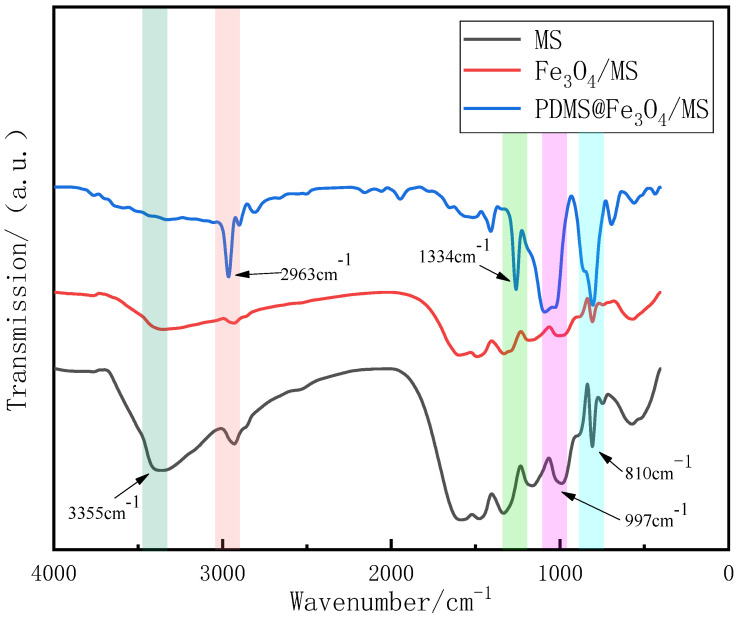
FTIR diagrams of MS, Fe_3_O_4_/MS and PDMS@Fe_3_O_4_/MS (The shadows from left to right are 3355 cm^−1^, 2963 cm^−1^, 1334 cm^−1^, 997 cm^−1^ and 810 cm^−1^).

According to the FTIR diagram of PDMS@Fe_3_O_4_/MS in Figure 6, we noticed that there is a strong symmetrical C-H stretching vibration peak of -CH_3_ at 2963 cm^−1^. The appearance of this characteristic peak indicates that PDMS has covered the sponge skeleton. At the same time, because the number of oxygen-containing groups on the surface of PDMS@Fe_3_O_4_/MS is reduced sharply at this time, the hydrophobicity and lipophilicity of the PDMS@Fe_3_O_4_/MS are improved. The rapid decrease in the number of oxygen-containing groups on the surface of PDMS@Fe_3_O_4_/MS is caused by PDMS coverage. This is very beneficial for improving the hydrophobicity and lipophilicity of the PDMS@Fe_3_O_4_/MS. It is worth noting that, compared with MS, the peak of PDMS@Fe_3_O_4_/MS at 3355 cm^−1^ almost disappears. This is also a sign of a significant reduction in oxygen-containing functional groups. This change promotes the hydrophobicity of the material and its performance is improved to a certain extent. Furthermore, the peaks at 810 cm^−1^ and 1334 cm^−1^ can be judged to be triazine ring bending vibration peaks of the triazine ring of MS [28,29] besides the peaks at 997 cm^−1^ are the C-H bending vibration peaks.

### 3.2. Modified Sponge Oil Absorption Capacity and Oil-Water Separation Test

#### 3.2.1. Oil Absorption Capacity of Modified Sponge

**Figure 7:**
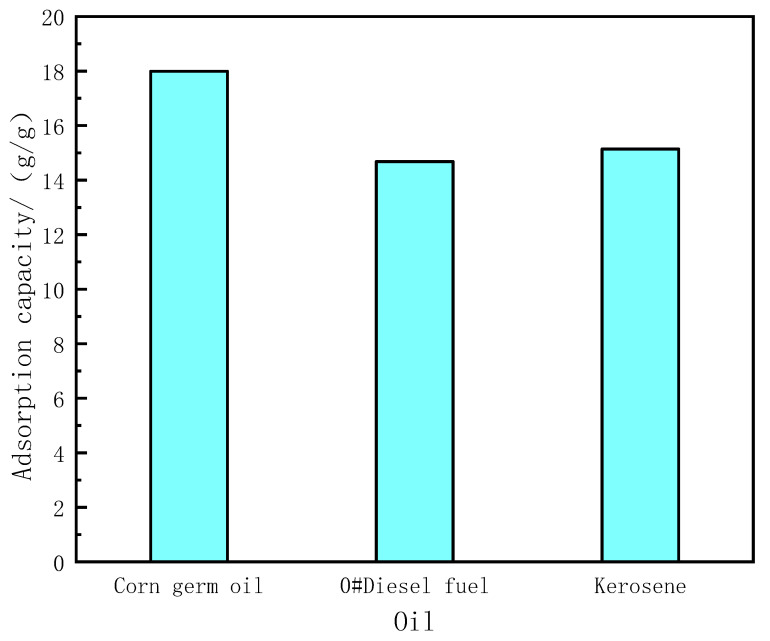
The oil absorption capacity of PDMS@Fe_3_O_4_/MS for different oils.

Through formula (1), we calculated the adsorption capacity of PDMS@Fe_3_O_4_/MS for the three oil products. The adsorption capacity of PDMS@Fe_3_O_4_/MS for corn germ oil is 17.99 ± 0.26 g/g; the adsorption capacity of 0# diesel is 14.68 ± 0.21 g/g; the adsorption capacity of kerosene is 15.14 ± 0.38 g/g. The experimental results are shown in Figure 7. In view of the low adsorption capacity of PDMS@Fe_3_O_4_/MS, we conducted comparative experiments with MS (10 mm × 10 mm × 10 mm) and PDMS@Fe_3_O_4_/MS (10 mm × 10 mm × 10 mm). The experimental results are shown in Table 1. According to the information in the table, We can find that the initial mass difference between PDMS@Fe_3_O_4_/MS and MS is more than 6 times at the same volume, but their adsorption capacity for 0# diesel is almost the same. This indicates that hydrophobic modification will increase the weight of the sponge, but will not significantly affect the open volume of the sponge’s pores. As long as the pore volume of the sponge is not significantly reduced, the amount of grease absorbed by the sponge will not decrease. Therefore, the adsorption capacity of PDMS@Fe_3_O_4_/MS is great well.

#### 3.2.2. Modified Sponge Oil-Water Separation Test

**Figure 8:**
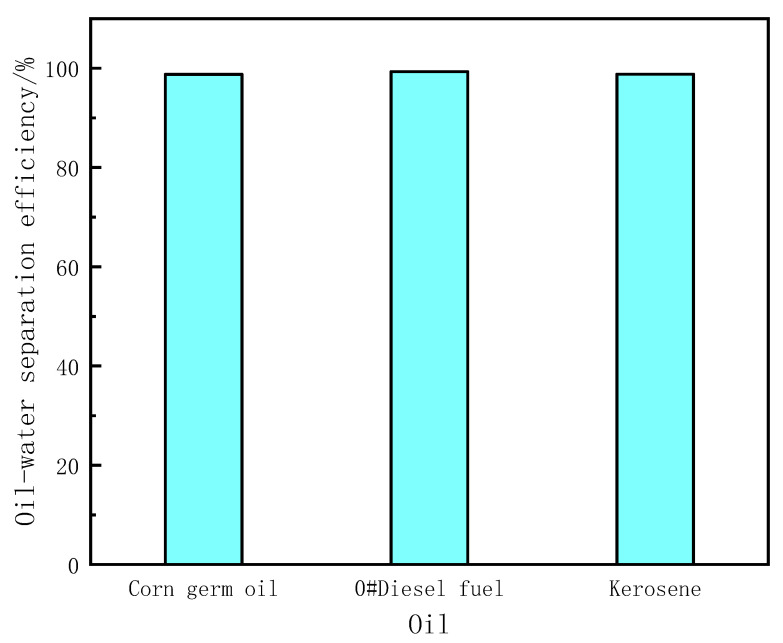
Separation efficiency of PDMS@Fe_3_O_4_/MS for different oil-water mixtures.

In Figure 8, the oil-water separation efficiency of PDMS@Fe_3_O_4_/MS for corn germ oil/water mixture, 0#diesel/water mixture and kerosene/water mixture are 98.78 ± 0.023%, 99.34 ± 0.045% and 98.80 ± 0.019%. This is the result calculated according to formula (2). This efficient oil-water separation performance is determined by the three-dimensional porous structure of MS and the excellent hydrophobic properties of the PDMS coating.

#### 3.2.3. Modified Sponge Repeated Oil Absorption and Oil-Water Separation Performance Test

**Figure 9:**
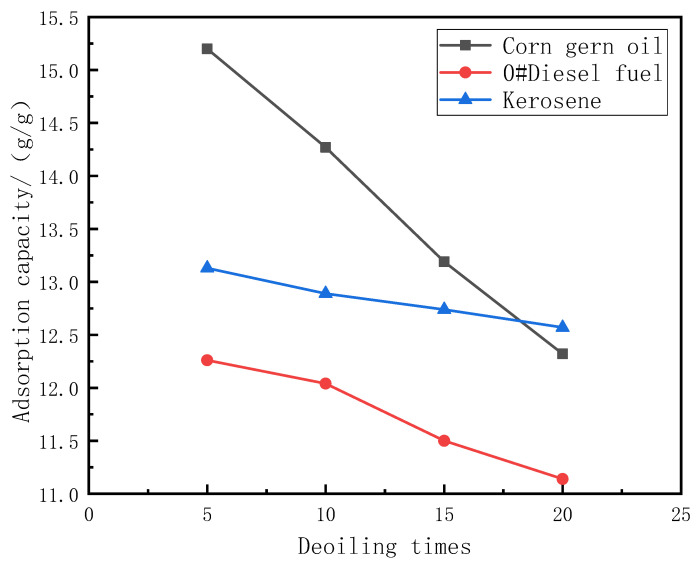
Adsorption capacity of PDMS@Fe_3_O_4_/MS after multiple cycles.

**Figure 10:**
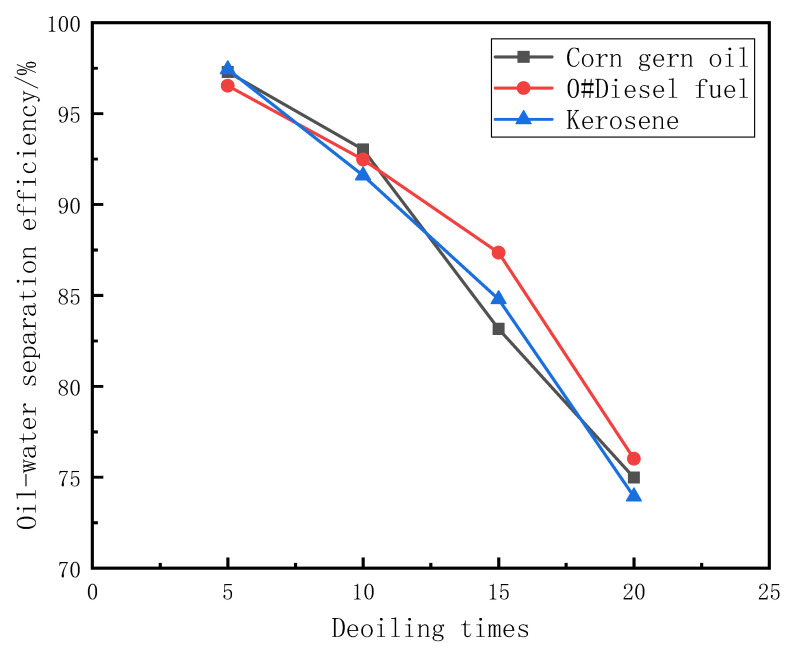
Oil-water separation efficiency after multiple cycles of PDMS@Fe_3_O_4_/MS.

The mechanical squeeze method is used to measure the reusability of PDMS@Fe_3_O_4_/MS. The PDMS@Fe_3_O_4_/MS is used to repeatedly absorb and deoil the oil-water mixture of corn germ oil, 0#diesel oil, kerosene and three kinds of oil products. The adsorption capacity and oil-water separation efficiency were measured after a certain number of oil absorption and deoiling. We measured the adsorption capacity and oil-water separation efficiency of PDMS@Fe_3_O_4_/MS after deoiling 5 times, 10 times, 15 times and 20 times. Table 2 shows the adsorption capacity of PDMS@Fe_3_O_4_/MS after deoiling 5 times, 10 times, 15 times and 20 times. Table 3 shows the oil-water separation efficiency of PDMS@Fe_3_O_4_/MS after deoiling 5 times, 10 times, 15 times, and 20 times. Figure 9 is a graph showing changes in adsorption capacity of PDMS@Fe_3_O_4_/MS after repeated oil absorption and deoiling. Figure 10 is a graph showing the change in oil-water separation efficiency of PDMS@Fe_3_O_4_/MS after repeated oil absorption and deoiling.

**Figure 11:**
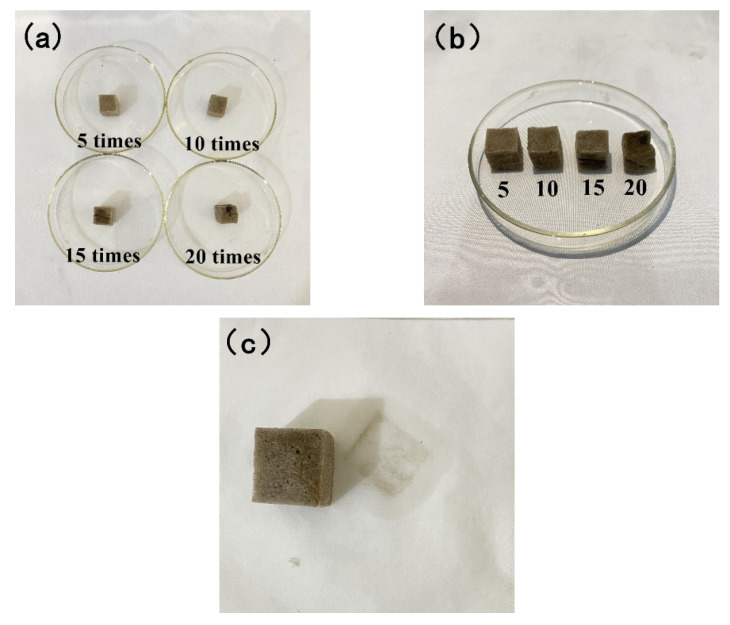
(**a**,**b**) the appearance of PDMS@Fe_3_O_4_/MS after deoiling 5 times, 10 times, 15 times, and 20 times and (**c**) The paper towel absorbs the residual oil in the sponge.

After PDMS@Fe_3_O_4_/MS undergoes multiple oil absorption-deoil cycles, the oil absorption capacity and oil-water separation efficiency are reduced. There are two main reasons for this phenomenon. First of all, the method of mechanical extrusion severely damaged the structure of the sponge. The morphology of the sponge has undergone significant changes after repeated oil absorption and deoiling. The elasticity of the sponge begins to weaken at the same time. Mechanical squeezing made it lose the ability to restore its original shape. Figure 11a,b shows the appearance of PDMS@Fe_3_O_4_/MS after deoiling 5 times, 10 times, 15 times, and 20 times. Secondly, the mechanical squeeze method cannot completely squeeze the oil inside the sponge. There is bound to be a slight oil residue inside the sponge. Figure 11c shows that the paper towel absorbs the residual oil of PDMS@Fe_3_O_4_/MS.

### 3.3. Test of Modified Sponge Affected by Magnetic Force

**Figure 12:**
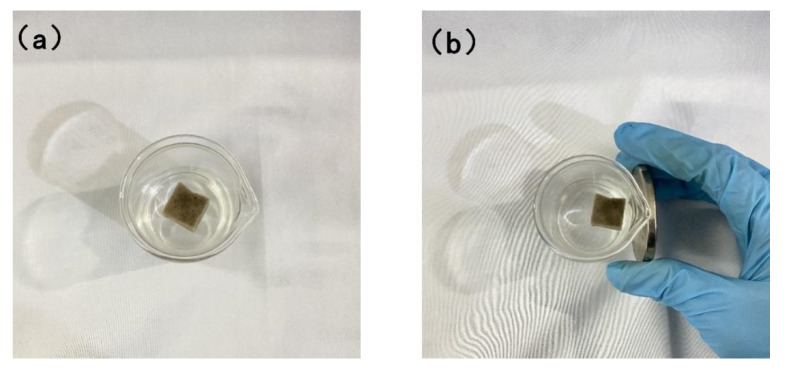
(**a**) PDMS@Fe_3_O_4_/MS is at rest on the water surface and (**b**) PDMS@Fe_3_O_4_/MS is close to the cup wall under magnetic force.

Put the superhydrophobic melamine sponge into a beaker with a certain amount of water and the sponge will be suspended on the surface of the water. With a magnet slowly approaching the beaker, the nano-Fe_3_O_4_ [30,31] on the surface and inside of the sponge drives the sponge to move rapidly from the middle of the beaker to the side of the beaker under the action of magnetic force, as shown in Figure 12. This feature allows PDMS@Fe_3_O_4_/MS to move according to whether the oil stains in the area have been absorbed. After the oil-water separation in one area is completed, the sponge is driven to move to other oil-bearing areas by magnetic force. In this way, the sponge can separate oil and water more efficiently. Moreover, the most important thing is that it can be recycled with the help of magnetism. The recycled sponge can be reused after being mechanically squeezed, which can greatly reduce the cost of oil-water separation.

## 4. Conclusions

This article is based on the melamine sponge through the oxidative polymerization of dopamine hydrochloride, nano-Fe_3_O_4_ adsorption and PDMS modification to obtain superhydrophobic magnetic melamine sponge. This superhydrophobic magnetic melamine sponge has a three-dimensional porous structure with a PDMS coating on the surface. The PDMS coating makes the sponge have excellent superhydrophobicity. The test shows that the water contact angle is 150.9°, and the oil contact angle is 0°. The sponge has excellent oil absorption capacity and excellent oil-water separation ability, and can absorb oil products of more than 14 times its own mass, and the separation efficiency of oil-water mixture can be as high as 99.34% in a single time. The sponge also has excellent reusability. After 20 oil absorption and deoiling operations, the sponge can still absorb up to 12.57 times of its own oil, reaching an oil-water separation efficiency of more than 70%. In addition, nano-Fe_3_O_4_ can make the sponge magnetic. This feature allows the sponge to move regionally under the action of magnetic force to improve the efficiency of oil-water separation; on the other hand, the staff can also use the magnetic force to recycle the sponge. The large-scale application of PDMS@Fe_3_O_4_/MS in waste oil treatment and recycling is very worthy of promotion.

## Figures and Tables

**Table 1:** Comparison of adsorption capacity of MS and PDMS@Fe_3_O_4_/MS.

Sponge	Initial Mass/g	Mass after Adsorption/g	The Mass of Adsorbed Oil/g	Adsorption Capacity g/g
MS	0.03	2.81	2.79	93
PDMS@Fe_3_O_4_/MS	0.19	2.94	2.75	14.49

**Table 2:** Adsorption capacity of PDMS@Fe_3_O_4_/MS after multiple cycles.

Oil	5 Times (g/g)	10 Times (g/g)	15 Times (g/g)	20 Times (g/g)
Corn gern oil	15.20 ± 0.21	14.27 ± 0.25	13.19 ± 0.19	12.32 ± 0.23
0#Diesel fuel	12.26 ± 0.21	12.04 ± 0.24	11.50 ± 0.23	11.14 ± 0.22
Kerosene	13.13 ± 0.34	12.89 ± 0.31	12.74 ± 0.37	12.57 ± 0.35

**Table 3:** Oil-water separation efficiency after multiple cycles of PDMS@Fe_3_O_4_/MS.

Oil	5 Times (%)	10 Times (%)	15 Times (%)	20 Times (%)
Corn gern oil	97.28 ± 0.023	93.02 ± 0.021	83.16 ± 0.029	74.98 ± 0.027
0#Diesel fuel	96.53 ± 0.041	92.47 ± 0.039	87.35 ± 0.041	76.02 ± 0.037
Kerosene	97.43 ± 0.020	91.60 ± 0.022	84.79 ± 0.018	73.94 ± 0.024

## Data Availability

The raw/processed data required to reproduce these findings cannot be shared at this time as the data also forms part of an ongoing study.
